# Evaluation of hydroxyapatite and beta-tricalcium phosphate mixed with bone marrow aspirate as a bone graft substitute for posterolateral spinal fusion

**DOI:** 10.4103/0019-5413.49387

**Published:** 2009

**Authors:** Sanjay Bansal, Vijendra Chauhan, Sansar Sharma, Rajesh Maheshwari, Anil Juyal, Shailendra Raghuvanshi

**Affiliations:** Department of Orthopaedic Surgery, Himalayan Institute of Medical Sciences, Swami Ram Nagar, Jollygrant, Doiwala, Dehradun- Uttarakhand-248 140, India; 1 Department of Radiodiagnosis, Himalayan Institute of Medical Sciences, Swami Ram Nagar, Jollygrant, Doiwala, Dehradun- Uttarakhand-248 140, India

**Keywords:** Beta-tricalcium phosphate, bone marrow aspirate, hydroxyapatite, posterolateral spinal fusion

## Abstract

**Background:**

Autologous cancellous bone is the most effective biological graft material. However, harvest of autologous bone is associated with significant morbidity. Since porous hydroxyapatite and beta-tricalcium phosphate are biodegradable materials and can be replaced by bone tissue, but it lacks osteogenic property. We conducted a study to assess their use as a scaffold and combine them with bone marrow aspirate for bone regeneration using its osteogenic property for posterolateral spinal fusion on one side and autologous bone graft on the other side and compare them radiologically in terms of graft incorporation and fusion.

**Materials and Methods:**

Thirty patients with unstable dorsal and lumbar spinal injuries who needed posterior stabilization and fusion were evaluated in this prospective study from October 2005 to March 2008. The posterior stabilization was done using pedicle screw and rod assembly, and fusion was done using hydroxyapatite and beta-tricalcium phosphate mixed with bone marrow aspirate as a bone graft substitute over one side of spine and autologous bone graft obtained from iliac crest over other side of spine. The patients were followed up to a minimum of 12 months. Serial radiographs were done at an interval of 3, 6, and 12 months and CT scan was done at one year follow-up. Graft incorporation and fusion were assessed at each follow-up. The study was subjected to statistical analysis using chi-square and kappa test to assess graft incorporation and fusion.

**Results:**

At the end of the study, radiological graft incorporation and fusion was evident in all the patients on the bone graft substitute side and in 29 patients on the autologous bone graft side of the spine (*P* > 0.05). One patient showed lucency and breakage of distal pedicle screw in autologous bone graft side. The interobserver agreement (kappa) had an average of 0.72 for graft incorporation, 0.75 for fusion on radiographs, and 0.88 for the CT scan findings.

**Conclusion:**

Hydroxyapatite and beta-tricalcium phosphate mixed with bone marrow aspirate seems to be a promising alternative to conventional autologous iliac bone graft for posterolateral spinal fusion.

## INTRODUCTION

Autologous cancellous bone is the most effective biological graft material, since it possesses the three core properties: osteoconduction, osteoinduction, and osteogenic cells.^1^,^2^ However, harvest of autologous bone is associated with significant morbidity.^3^–^5^

To overcome these problems, biological alternatives, mainly allografts and xenografts, have been processed and used. However, limitation of easy availability, high cost, and problems of immunogenicity have accelerated the search for synthetic, bioinert materials as an alternative.^6^

Hydroxyapatite [Ca_10_(PO_4_)_6_(OH)_2_] and beta-tricalcium phosphate [Ca_3_(PO_4_)_2_] ceramics are biocompatible and osteoconductive materials that offer a chemical environment and a surface conducive to new bone formation.^7^–^10^ These are brittle materials and have low fracture resistance.^11^ Different preparative methods lead to either a compact or porous material with interconnective macropores that are spatially and structurally equivalent to cancellous bone.^12^ Commercially available hydroxyapatite is resorbed very slowly, under normal physiological conditions, whereas beta-tricalcium phosphate is generally resorbed within 6 weeks after implantation.^13^ When used as a mixture, biodegradable hydroxyapatite/beta-tricalcium phosphate ceramics have the ability to dissolve, break down, and allow new bone formation and remodeling required to attain optimal mechanical strength without interference. A fully nonresorbable graft material may hinder remodeling, cause strength deficiency of new bone and leave permanent stress risers in the fusion mass.^14^ The acceptance of these substitutes by host tissues is determined by two important features: pore diameter and the porosity or interconnectivity. Minimum pore size of 100 *μ*m is optimal for bone in growth where as pore size more than 200 *μ*m facilitates development of mature osteon.^15^

Bone marrow harvested by aspiration contains osteoblastic progenitors with other bone marrow-derived cells that are rich in cytokines. It also provides a degradable biologic matrix of fibrin, which can revascularize rapidly.^16^,^17^

Hence, we conducted a study to asses the use of hydroxyapatite and beta-tricalcium phosphate mixed with bone marrow aspirate as a bone graft substitute for posterolateral spinal fusion in cases of spinal fractures and compared it radiologically with autologous bone graft in the terms of graft incorporation and fusion.

## MATERIALS AND METHODS

This prospective study was carried out from October 2005 to March 2008 after obtaining approval from the Institutional Research Committee and informed consent from the patients. The study included 30 patients of which 22 were men and 8 were women; the mean age was 39.2 years (range, 18-55 years). Patients with unstable dorsal and lumbar spinal injuries that needed stabilization and fusion were included in the study. In 16 patients L_1_ vertebra was injured, whereas 5 patients each had L_2_ and D_11_ level injury and one patient each had D_12_, D_7-8_, D_10-11_, D_12_-L_1_ injury.

Patients who were unfit for general anesthesia, had ankylosing spondylitis, previous failed fusion in dorsal and lumbar region of spine, multiple myeloma, osteomalacia, severe osteoporosis, infections of the spine, immature skeleton, bleeding disorders, patient on anticoagulant therapy, radiation therapy, immunosuppressive drugs, severe anemic patient having Hb < 10 g%, disorders of bone marrow, and previous surgery on iliac crest to obtain bone graft were excluded from the study.

All the patients were operated in a prone position on a radiolucent table, keeping the abdomen free on bolsters. A midline longitudinal incision was made over the spinous process extending from two spinous processes above and two below the level of injury. In the lumbar spine, lamina decortication was done, including the excision of posterior third of inferior and superior articular process, cutting across facet joint. In the thoracic spine, distal third of inferior articular process was excised, exposing distal half of facet joint at each level on each side of spine.

The decortication included the lamina out to the tip of transverse process and the exposed portion of superior articular process on either side. Following decortication, pedicles of the above and below the injured vertebrae were identified using image intensifier and spinal column was stabilized using titanium pedicle screw and rod system.

Bone graft substitute was prepared in the following manner: 90% hydroxyapatite and 10% beta-tricalcium phosphate (bonograft), 6 g, having macroporosity of >300 *μ*m, was mixed with 4 ml of sterile saline and was allowed to hydrate for 1-3 minutes. About 4-5 ml of bone marrow aspirate obtained from the iliac crest using bone marrow needle was mixed properly with bonograft and was placed over the prepared decorticated bed over one side of the spine.

Autologous iliac graft slivers obtained from the opposite iliac crest were placed on the other side of the spine on the prepared bed. Incision was closed in layers.

Patients were mobilized on their beds with the help of Taylor's spinal brace from third postoperative day, and stitches were removed on twelfth postoperative day.

Plain radiographs were done at an interval of 3, 6, and 12 months. Graft incorporation was evaluated as per Irwin's radiological staging [Table 1].^18^ Fusion mass was considered good when there was a continuous block of bone without radiolucent areas; fusion mass was considered poor when no intersegmental bridging fusion was seen.

**Table 1:** Irwin's radiological stages of graft incorporation^18^

Stage	Radiological finding
I	Obvious margins
II	Hazy margins
III	Obvious incorporation

CT scans were done at one year follow-up. The radiological and CT scan findings were recorded by two independent radiologists. The observations thus obtained were subjected to statistical analysis, using SPSS software. Interobserver agreement was measured by the kappa test.

## RESULTS

The results are evaluated under following heads.

### 1. Roentgenogram evaluation

**Figure 1:**
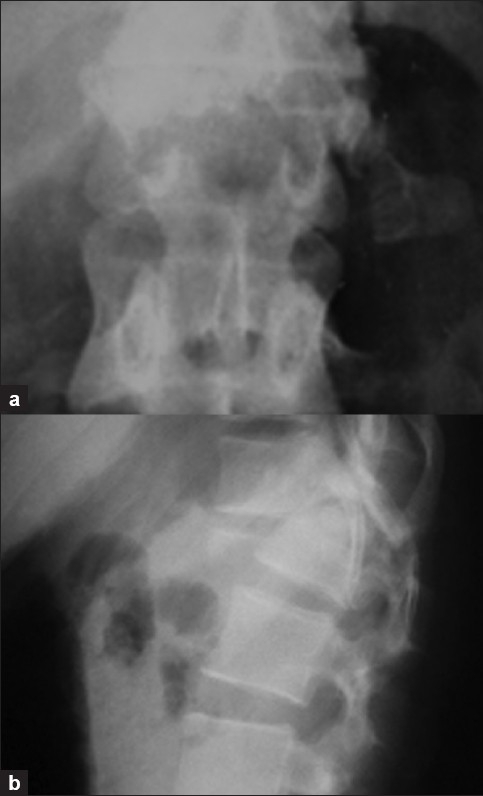
Preoperative X-ray [anteroposterior view (a) and lateral view (b)] of dorsolumbar junction showing fracture L1 vertebra

**Figure 2:**
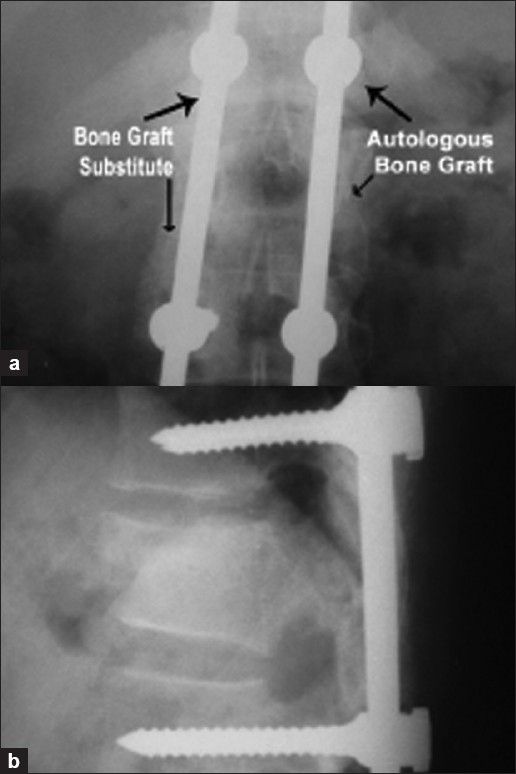
Six-month follow-up X-ray [anteroposterior view (a) and lateral view (b)] of the same patient showing pedicle screw and rod assembly *in situ* with fusion in progress on both the sides

**Figure 3:**
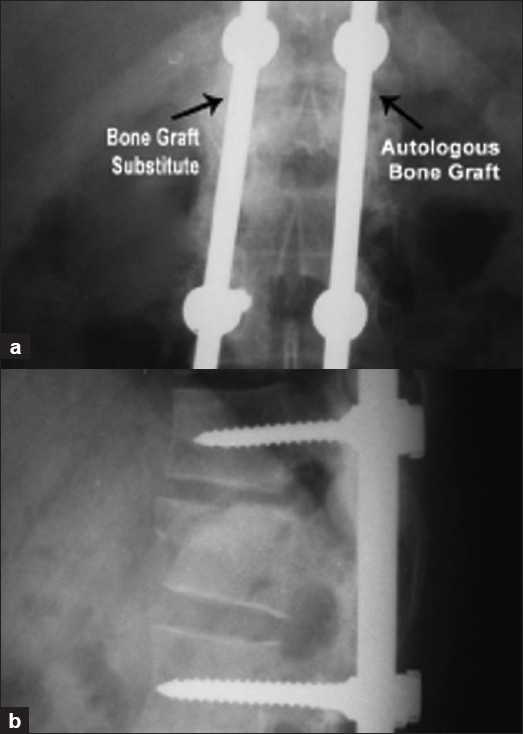
One year follow up X-ray [anteroposterior (a) and lateral view (b)] of the same patient showing bridging bone mass with graft incorporation on both the sides

The following observations were made by two independent radiologists [Figures 1–3].

#### a) Graft incorporation

##### Autologous bone graft side

Graft could be seen in situ in all the patients. Irwin's stage II graft incorporation was seen in all the patients at the end of 3 months. At the end of 6 months, the Irwin's stage lll graft incorporation was seen. At one year, better consolidation could be appreciated in 29 patients (*P* > 0.05). One patient had areas of lucency in the incorporated mass at 1 year. This patient had breakage of distal screw on same side. The implant was removed and biopsy was obtained from both the sides. Peroperatively, there was no sign of infection. On the other side (bone graft substitute side), good fusion mass could be seen. The biopsy revealed fibrous tissue from the autologous side and new bone formation on the bone graft substitute side. Patient was asymptomatic thereafter and was followed up further for 6 months.

##### Bone graft substitute side

Margins of the granules appeared to be slightly hazy (Irwin's stage II), and at 6-month follow-up, there was obvious incorporation (Irwin's stage III). However, the granules could still be identified. At the end of 1 year, granules could no longer be appreciated, and it became a homogenous mass in all the patients (*P* > 0.05).

The interobserver agreement (kappa) had an average of 0.72 for graft incorporation.

#### b) Fusion

##### Autologous bone graft side

Bridging bone mass could be appreciated in all the patients at the end of third month; became more prominent in all the patients at 6-month follow-up, and good fused mass could be appreciated in 29 patients at the end of 1 year (*P* > 0.05). Poor fusion mass was seen in one patient, who had lucencies and implant failure.

##### Bone graft substitute side

Bridging bone mass started appearing in all the patients at the end of third month, became more prominent in all the patients at 6-month follow-up, and a well-fused bridging bone mass could be appreciated in all the patients at the end of 1 year.

The interobserver agreement (kappa) had an average of 0.75 for the fusion masses.

### 2. CT scan observations

**Figure 4:**
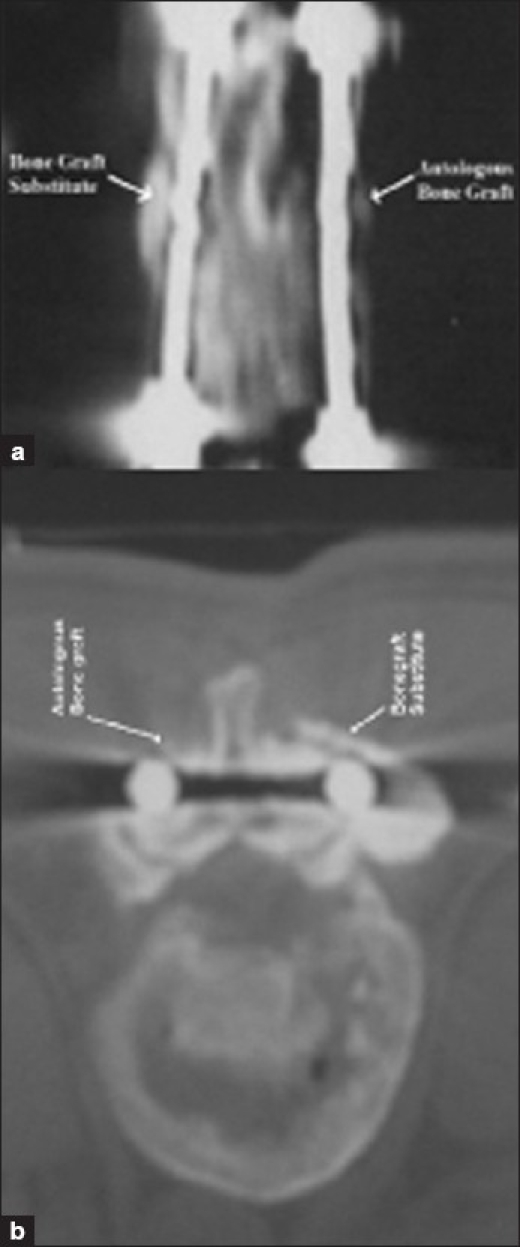
(a) CT scan of the same patient at one year followup showing evidence of good fusion on both sides. (b) CT scan axial cut of the same patient showing good fusion

CT scan was done in 30 patients at 1 year following surgery, and good fusion mass was seen in 29 patients on the autologous bone graft side and 1 patient showed lucencies in the graft. This patient had an implant breakage of the distal screw. On the other hand, all the 30 patients on the bone graft substitute side had good fusion mass [Figure 4].

The interobserver agreement (kappa) had an average of 0.88 for the CT scan findings.

### 3. Histological observations

**Figure 5:**
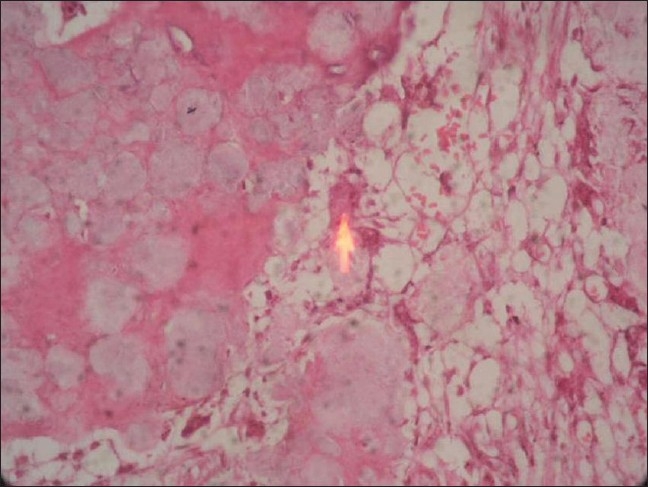
Photomicrograph of biopsy of bone graft substitute side stained with H & E (x100), showing evidence of new bone formation

**Figure 6:**
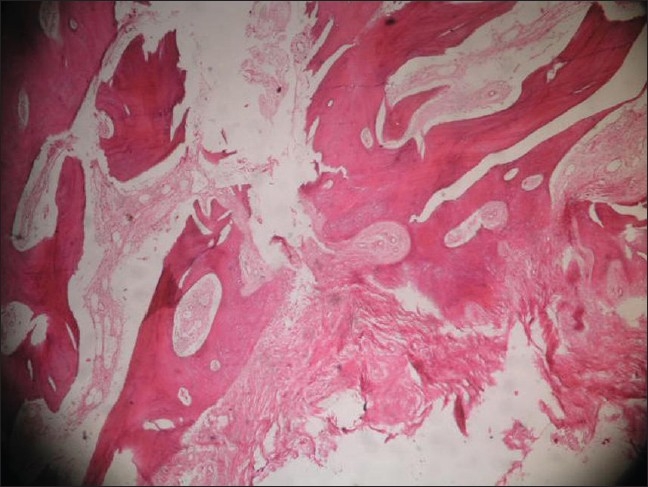
Photomicrograph of biopsy of the patient who had implant breakage and radiographic lucency on the autologous bone graft side, stained with H & E (x100), showing fibrous tissue formation with no evidence of new bone formation

The fused mass was biopsied in three patients from both the sides, at the time of elective removal of the implant after 2 years of follow-up in two patients and one in which the implant had failed at one year. It was observed that on the bone graft substitute side, many bony trabeculae of various thicknesses that were very cellular and showed lamellar pattern were formed. Amidst these, collections of foamy substitute materials of variable sizes with new bone formation in their substances were seen and were surrounded by osteoblasts and osteoclasts [Figure 5]. Two patients on autologous bone graft side showed new bone formation and the patient with implant failure showed fibrous tissue formation in the areas of radiological lucencies [Figure 6]. Since only three patients consented for the biopsy, the observation group is considered statistically insignificant.

### 4. Complications

Five patients had donor site pain, and one had sensory loss on the lateral aspect of thigh. As previously mentioned, one patient had implant breakage. None of the patients showed graft rejection or developed infection. The final results are as shown in Table 2.

**Table 2:** Final observations at the end of 12 months follow-up

Observations	Bone graft substitute side (No. of patients)	Autologous bone graft side (No. of patients)
Stage III graft	30	30
Incorporation		
Good fusion	30	29
Poor fusion	0	1
Complications		
Implant breakage	0	1
Donor site pain	–	5
Sensory loss over lateral aspect of thigh	–	1

## DISCUSSION

Although osteoconduction and osteoinduction must be present in any successful graft site, no osteoconductive material or osteoinductive stimulus will be effective in the absence of osteogenic cells. Osteogenic cells may be transplanted into the graft by an autologous graft of bone or bone marrow. They also may migrate into the graft site from surrounding tissues, or be delivered to the graft site through the vascular system during and after revascularization. Regardless of the pathway, all successful graft procedures require some minimal number of osteogenic progenitors within the graft site.^19^

Calcium phosphate ceramics, whose composition is similar to that of mineral bone, are known to be safe and nonallergic, with good bone bonding capacity. They have been explored and used as substitute for bone graft in orthopedics, maxillofacial, and dental operations over the last three decades. Hydroxyapatite is generally considered to be only slightly resorbable, whereas beta-tricalcium phosphate is resorbed by a cellular pathway, leaving newly formed bone tissue. The different resorption rates of these two constituents provide a diminishing scaffold for bone formation and orderly remodeling over the time. Hydroxyapatite has minimal resorbability and acts as a scaffold for bone ingrowth by providing a fixed structure for calcification to occur.^20^,^21^ When used as a combination of hydroxyapatite and beta-tricalcium phosphate, it has the ability to dissolve, break down, and allow new bone formation and remodeling required to attain optimal mechanical strength without interference.^14^

Since porous hydroxyapatite and beta-tricalcium phosphate are biodegradable materials and can be replaced by bone tissue, we used them as a scaffold and combined with bone marrow aspirate for bone regeneration using its osteogenic property and thus utilized bone marrow aspirate, hydroxyapatite, and beta-tricalcium phosphate as a composite graft. Composite grafts of this nature have been used experimentally in animal studies, and few human studies have shown good results.^22^–^27^

We observed that graft incorporation could be appreciated radiologically by third month on bone graft substitute side, and over the autologous bone graft side, and by the end of one year the graft became more consolidated on both the sides. The granules had almost merged and formed a dense mass over the bone graft substitute side. This density was more in comparison to the autologous bone graft side. The reason is that hydroxyapatite has a very slow resorption rate and thus keeps on casting its dense shadow on radiographs for a longer time.

Bridging bone mass could be seen from third month onward on bone graft substitute side and autologous bone graft side, and by the end of one year, the bone mass became more homogenous. Our results were comparable to those obtained by Luis *et al.*^28^

Although clinically and radiologically it appears that there is a progressive bony fusion, it is difficult to comment radiologically on the quality of fusion. Definitive criteria need to be laid down to assess fusion. The quality of fusion can only be assessed on histology, which is impracticable in human beings; as seen in our study, out of 30 patients only 2 patients underwent elective implant removal and biopsy. The third one was biopsied, since he required an implant removal because of implant failure. The histological findings that we obtained in these three cases are similar to those of Uchida *et al.*^29^ This we believe is the drawback of our study, as we could not correlate the radiological findings with histopathological findings.

A 12-months' time was chosen in the present study because it is a sufficient time to evaluate incorporation and fusion of the bone graft substitutes, as the current instrumentation requires about one year time to obtain a solid arthrodesis.^30^

We aspirated bone marrow, 1 ml each, from four to five different sites to get the maximum concentration of progenitor cells.^17^ Although optimum number of osteoprogenator cell required are obtained from 100 to 150 ml of bone marrow, such a large quantity not only may wash away from the fusion site as quoted by Christopher *et al.*,^31^ which also decreases the concentration of osteoprogenator cells due to dilution.^17^ We believe that osteoprogenator cells also seep in from the decorticated bed.^19^

From the above radiological results, it appears that hydroxyapatite and beta tricalcium phosphate mixed with bone marrow aspirate supports new bone growth and maturation and can be safely used for spinal fusion and is as effective as autologous bone graft with an added advantage of no donor site morbidity.
